# Genome-Wide Identification of the *CCCH* Gene Family and Functional Exploration of *MdC3H49* Under Drought Stress Response in Apple (*Malus domestica*)

**DOI:** 10.3390/plants15081270

**Published:** 2026-04-21

**Authors:** Da Zhang, Chao Zhao, Bowei Zhu, Xin Liu, Han Wang, Yaping Song, Guodong Zhao, Linguang Jia, Dongmei Chen, Tongsheng Zhao, Xinsheng Zhang, Chaohong Zhang

**Affiliations:** 1 Changli Institute of Pomology, Hebei Academy of Agriculture and Forestry Sciences, Qinhuangdao 066600, China; d.zhang@nwafu.edu.cn (D.Z.); 18331561549@163.com (B.Z.); 18830180711@163.com (X.L.); 15830298656@163.com (H.W.); syp0526@126.com (Y.S.); guodong19823@163.com (G.Z.); dsjialinguang2020@163.com (L.J.); chendm2009@126.com (D.C.); tshzh71@163.com (T.Z.); 2 State Key Laboratory for Crop Stress Resistance and High-Efficiency Production, Northwest A&F University, Yangling 712100, China; zhaochao123@nwafu.edu.cn; 3 Shijiazhuang Institute of Pomology, Hebei Academy of Agriculture and Forestry Sciences, Shijiazhuang 050051, China

**Keywords:** *CCCH* gene family, drought tolerance, *Malus domestica*, *MdC3H49*

## Abstract

CCCH zinc-finger proteins constitute a unique class of transcription factors that play vital roles in mediating plant tolerance to biotic and abiotic stresses and regulating various physiological and developmental processes. This study systematically identified and characterized the apple (*Malus domestica*) *CCCH* (*MdC3H*) gene family, aiming to elucidate its evolutionary patterns, functional characteristics, and regulatory mechanisms under drought stress. Genome-wide analysis revealed 85 *MdC3H* genes, which were unevenly distributed across chromosomes and exhibited significant differences in physiochemical properties, suggesting functional divergence. Phylogenetic analysis classified these genes into 9 subfamilies with distinct conservation. Collinearity analysis indicated a close evolutionary relationship between apple and *Malus sieversii*, with 150 collinear gene pairs identified, highlighting the conservation of the *C3H* gene family during speciation. Cis-acting element prediction in promoter regions uncovered abundant stress-responsive elements (e.g., ABRE, DRE, MYB), implying the potential of *MdC3H* genes in coordinating environmental signals. Functional verification demonstrated that *MdC3H49*, a key member of the family, is localized in the nucleus and possesses transcriptional activation activity. Overexpression of *MdC3H49* in *Arabidopsis* and apple calli significantly enhanced drought tolerance, characterized by reduced malondialdehyde (MDA) content, relative electrical conductivity, and increased proline accumulation. Mechanistic studies revealed that MdC3H49 directly regulates the expression of *MdP5CS*, a core gene in proline biosynthesis, thereby strengthening the cellular antioxidant capacity and mitigating drought-induced damage. Collectively, this study establishes MdC3H49 as a critical regulator in apple drought stress response, providing valuable insights into the molecular mechanisms underlying abiotic stress tolerance in perennial plants and laying a foundation for genetic improvement of drought resistance in apple breeding.

## 1. Introduction

Zinc-finger proteins are a class of sequence-specific transcription factors that usually contain varying numbers of cysteine (Cys) and histidine (His) residues. They are named for their ability to bind Zn^2+^, which stabilizes a short, self-folding “finger-like” structure [1,2,3]. As widely existing regulators in various organisms, C3H zinc-finger genes play crucial roles in plant development and stress responses through interactions with DNA, RNA, or proteins [4]. Zinc-finger proteins are classified into several types, including C2H2, C8, C6, C3HC4, C2HC5, C4, C4HC3, and CCCH, based on the number and position of conserved Cys and His residues [2]. CCCH zinc-finger proteins generally contain one or more zinc-finger motifs, with three Cys and one His residue being the most important components of this motif [5]. In addition, these CCCH proteins usually contain 1 to 6 CCCH-type zinc-finger motifs, while ZmC3H3 in maize contains 7 CCCH-type zinc-finger motifs [6]. According to the number of amino acid spacers between Cys and His residues in the CCCH motif, the consensus sequence of these motifs is defined as C-X_4-17_-C-X_4-6_-C-X_3-4_-H (where X represents any amino acid). In *Arabidopsis* [7] and *Oryza sativa* [8], the most abundant CCCH zinc-finger motifs are C-X_3_-C-X_5_-C-X_3_-H and C-X_7_-C-X_5_-C-X_3_-H, which are also the most common CCCH motifs in other plant species [1,9].

Zinc-finger proteins exhibit diverse functions in different developmental stages and tissues of plants [10,11]. CCCH zinc-finger proteins are involved in the regulation of plant development, adaptation, hormone regulation, and physiological stress-related processes, especially in the response to biotic and abiotic stresses [2]. Currently, the *CCCH* gene family has been extensively reported in various plant species, such as *Arabidopsis*, *Glycine max* [12], *Cucumis sativus* [13], *Morus alba* [14], and *Gossypium hirsutum* [15]. Abscisic acid (ABA) and salt stress can enhance plant tolerance to drought, oxidative, and salt stresses by inducing the expression of *Arabidopsis* CCCH zinc-finger proteins AtTZF1, AtTZF2, and AtTZF3 [16]. In *Medicago sativa*, overexpression of *MsC3H29* can increase the taproot length and fresh weight of transgenic alfalfa hairy roots, while RNA interference (RNAi) reduces these indicators under drought stress. MsC3H29 improves the drought tolerance of alfalfa hairy roots by reducing reactive oxygen species (ROS) accumulation and increasing the levels of flavonoids, including visinin, genistein, apigenin, isorhamnetin, quercetin, and tricin [17]. In buckwheat, FtCCCH56 enhances tolerance to drought and salt stresses by upregulating ABA-responsive genes (*AtRD29A*, *AtRD29B*, *AtRAB18*, *AtRD22*, *AtKIN1*, *AtCOR15A*) in transgenic *Arabidopsis*, increasing antioxidant enzyme activity, and promoting proline biosynthesis [18]. The *Glycine max* tandem CCCH zinc-finger protein gene *GmZF351* is regulated by histone demethylases GmJMJ30-1 and GmJMJ30-2. When soybeans are subjected to environmental stresses, the *GmZF351* gene is activated in roots and leaves, participates in stress tolerance responses, and improves the stress resistance of soybeans [19]. Nevertheless, the research on the C3H gene family remains to be further explored.

Apple (*Malus domestica*) is an important temperate deciduous fruit tree in China and one of the most widely cultivated economic fruit trees worldwide. Its yield and quality are easily severely affected by drought stress [20,21]. Therefore, in-depth analysis of the molecular defense mechanisms of apple in response to drought stress is of great theoretical and practical significance for apple genetic improvement and stress-resistant breeding. C3H zinc-finger proteins play a key role in the molecular regulation of plant responses to abiotic stresses. However, the genome-wide identification, phylogenetic characteristics, and stress-resistant functional regulatory mechanisms of the apple *C3H* gene family have not been systematically and in-depth studied. Therefore, based on the publicly available apple reference genome data, this study intends to comprehensively identify the members of the *C3H* gene family at the whole-genome level of apple using bioinformatics methods. We will systematically analyze the evolutionary relationships, gene structures, conserved motifs, promoter *cis*-acting elements, and protein–protein interaction relationships of this family. Meanwhile, the expression patterns of typical members of the *MdC3H* family under drought stress will be analyzed byRT-qPCR. The drought-resistant function will be verified by transforming *Arabidopsis* and apple *callus*. In addition, we will verify the regulatory effect of MdC3H49 on the *MdP5CS* gene using yeast one-hybrid (Y1H) and dual-luciferase (Dual-LUC) assays. This study aims to provide a theoretical basis for in-depth analysis of the biological functions of apple *C3H* genes, as well as important candidate gene resources and a molecular breeding foundation for the selection and breeding of new stress-resistant apple varieties.

## 2. Materials and Methods

### 2.1. Genome-Wide Identification of Apple C3H Family Members

To accurately identify the *C3H* family members in the apple genome, the whole-genome protein sequences of the apple cultivar ‘Golden Delicious’ were downloaded from the Ensembl Plants database (https://plants.ensembl.org/index.html (accessed on 5 July 2025), Genome assembly: ASM211411v1). The Hidden Markov model (HMM) profile of the C3H domain (Pfam ID: PF01167) was retrieved from the Pfam database (http://pfam.xfam.org/ (accessed on 29 June 2025)). HMMER software (v3.3.2) was employed to predict candidate C3H proteins in the apple genome with a default E-value cutoff. Additionally, a local BLASTP search (E-value < 0.001) was performed against the apple proteome using *Arabidopsis* C3H protein sequences as queries in TBtools software (v2.056). The candidate sequences obtained from both the HMM scan and BLASTP search were merged and deduplicated to define the final set of apple C3H candidates (Table S1). To ensure the reliability of the predicted sequences, the conserved domains of these candidate proteins were further verified using the online tools SMART (Simple Modular Architecture Research Tool, http://smart.embl.de/ (accessed on 10 May 2025)) and Batch CD-Search in the NCBI (https://www.ncbi.nlm.nih.gov/Structure/bwrpsb/bwrpsb.cgi (accessed on 10 May 2025)). Conserved motif analysis was conducted on the MdC3H protein sequences using the MEME online server (http://meme-suite.org/ (accessed on 25 May 2025)) with the maximum number of motifs set to 9. The *MdC3H* genes were named sequentially based on their chromosomal positions. The molecular weight (MW) and isoelectric point (pI) of the MdC3H proteins were calculated using the ExPASy ProtParam tool (https://web.expasy.org/protparam/ (accessed on 10 June 2025)).

### 2.2. Phylogenetic Tree Construction

The C3H protein sequences from *Arabidopsis*, *Actinidia chinensis*, *Pyrus bretschneideri*, and *Citrus sinensis* were identified using the same pipeline described above. A total of 66, 81, 66, and 70 C3H proteins were identified from these species, respectively. These sequences, together with the 85 newly identified MdC3H proteins from apple, were aligned using ClustalW (http://www.ebi.ac.uk/clustalw/ (accessed on 25 October 2025)) with default parameters. A maximum likelihood (ML) phylogenetic tree was constructed using MEGAX (v10.1.8) [22] with the JTT+G+I model and 1000 bootstrap replicates. The resulting tree was visualized and optimized using the iTOL online platform (https://itol.embl.de/ (accessed on 25 October 2025)).

### 2.3. Chromosomal Localization and Synteny Analysis

The chromosomal location of *MdC3H* genes was mapped onto the apple chromosomes using the Gene Location Visualize From GTF/GFF tool in TBtools (v2.056) [23] based on the GFF3 annotation file. Genomic sequences and GFF3 annotation files of *Solanum lycopersicum* were downloaded from the Sol Genomics Network (https://solgenomics.net/ (accessed on 25 October 2025)). Genomic data and GFF3 annotations for *Arabidopsis* were obtained from The *Arabidopsis* Information Resource (TAIR, https://www.arabidopsis.org/ (accessed on 25 October 2025)). Genomes and GFF3 files for *A. chinensis*, *Vitis vinifera*, and *Malus sieversii* were acquired from the Ensembl Plants database (https://plants.ensembl.org/index.html (accessed on 25 October 2025)). To analyze syntenic relationships, the OneStep MCScanX [24] plugin in TBtools (v2.056) was used to detect collinear blocks between apple and the other four species. The generated output files containing chromosomal positions, gene pairs, and collinear blocks were visualized using the Advanced Circos function in TBtools (v2.056) [25].

### 2.4. Cis-Acting Elements Analysis and GO Annotation

According to the apple genome annotation, the 2000 bp upstream genomic sequences (promoter regions) of 85 *MdC3H* genes were extracted in batch using TBtools (v2.056). These promoter sequences were submitted to the PlantCARE online server (http://bioinformatics.psb.ugent.be/webtools/plantcare/html/ (accessed on 29 October 2025)) for the prediction of *cis*-acting elements. The results were statistically analyzed using Excel 16.0, and a heatmap was generated using R software (v4.3.0). For GO annotation, the whole-genome protein sequences of apple were first submitted to the eggNOG database (http://eggnog6.embl.de/ (accessed on 25 October 2025)), and the eggNOG-mapper v2 tool (parameter settings: default Diamond alignment mode, with the E-value < 1 × 10^−5^) was used to perform homologous alignment and functional annotation of the protein sequences. Subsequently, the ggplot2 package in R software (v4.3.0) was adopted to collate the GO annotation results and draw the GO classification statistics charts, respectively.

### 2.5. Material Treatment, RNA Extraction andRT-qPCR

Apple seedlings of the cultivar ‘Gala’ were used as plant materials [26]. Apple seedlings with uniform growth and 4–6 true leaves were pre-cultured (25 ± 2 °C, 16 h light/8 h dark, 60–70% relative humidity) for 1 week. The seedlings were foliar-sprayed with 100 μmol/L ABA or 100 μmol/L MeJA (both containing 0.05% ethanol) [27], with water containing equal ethanol as control. Each treatment had three biological replicates. After treatment, samples were immediately frozen in liquid nitrogen and stored at −80 °C for gene expression and physiological analysis.

Drought stress was simulated by treating seedlings with 15% PEG6000 (25 ± 2 °C, 16 h light/8 h dark) [28]. All treatments included three biological replicates. The leaves were collected at 0, 6, 12, 24, 48, 96, and 120 h post-treatment, with no fewer than 3 samples collected per time point. The leaves were immediately frozen in liquid nitrogen and stored at −80 °C. Total RNA was extracted using the Universal Plant RNA Extraction Kit (Huayueyang, Beijing, China, #401) following the manufacturer’s instructions. First-strand cDNA was synthesized from 1 μg of total RNA using the RevertAid First Strand cDNA Synthesis Kit (Thermo Fisher Scientific, Waltham, MA, USA, #K1162). Specific primers were designed using Primer3plus (https://www.primer3plus.com/index.html (accessed on 25 January 2026)) and synthesized by Sangon Biotech (Shanghai, China). The sequences of theRT-qPCR primers are listed in Table S2. RT-qPCR was performed using the HamQ Universal SYBR qPCR Master Mix (Vazyme, Nanjing, China, Q711) on a CFX96 Touch Real-Time PCR Detection System. The relative expression levels were calculated using the 2^−ΔΔCt^ method [29].

### 2.6. Subcellular Localization

The coding sequence (CDS) of *MdC3H49* was amplified by PCR using gene-specific primers (Table S2). The PCR product was purified and cloned into the linearized pCAMBIA-1302-GFP vector (digested with Spe I and Nco I) via seamless cloning (37 °C for 30 min, Vazyme, C112), generating the fusion construct pCAMBIA-1302-GFP-MdC3H49. The recombinant plasmid was transformed into *E. coli* DH5α competent cells by heat shock (42 °C for 1 min). Positive clones were screened on Luria–Bertani (LB) agar plates containing 50 μg·mL^−1^ kanamycin and verified by PCR and DNA sequencing. The correct construct was then transformed into *Agrobacterium tumefaciens* GV3101 via the freeze–thaw method [30]. The transformed bacteria were cultured overnight at 28 °C with shaking at 220 rpm until the GV3101 reached OD600 = 0.5. The cells were collected, resuspended in infiltration buffer, and incubated in the dark for 2–3 h. Subsequently, needleless syringe infiltration was performed on tobacco leaves to achieve *Agrobacterium*-mediated transient expression. After 2–3 days of culture, the green fluorescent protein (GFP) signals were observed under a laser scanning confocal microscope (Olympus, Tokyo, Japan, FV3000).

### 2.7. Yeast One-Hybrid Assay

The sequence was designed based on the core binding motif (5′-GGGAGA-3′) reported for C3H transcription factors [31]. The core sequence (5′-GGGAGA-3′) was triplicated, synthesized, and inserted into the pHis2 vector to generate the bait construct. The bait vector was transformed into yeast strain Y187, and positive transformants were selected on SD/-Trp (Trp = 400 µM) plates. Autonomous activation testing was performed by spotting the transformants onto SD/-His/-Trp plates supplemented with gradient concentrations of 3-amino-1,2,4-triazole (3-AT, 50–100 mM) to determine the optimal concentration for suppressing background activity [32]. The prey construct pGADT7-MdC3H49 was transformed into the Y187 bait strain. The transformation efficiency was calculated by plating on SD/-Leu/-Trp plates. The remaining culture was spread onto SD/-His/-Leu/-Trp plates containing 80 mM 3-AT and incubated at 28–30 °C for 3–5 days. Positive colonies were identified by PCR and sequencing.

### 2.8. Dual-Luciferase Reporter Assay

The coding region of *MdC3H49* was cloned into the pGreenII 62-SK vector as an effector. The promoter fragment of *MdP5CS* was inserted into the pGreenII 0800-LUC vector as a reporter, with Renilla luciferase (REN) serving as an internal reference [31]. The reporter and effector plasmids were co-transfected into *Arabidopsis* protoplasts. After incubation in the dark, the protoplasts were collected, and the activities of firefly luciferase (LUC) and REN were measured using the Dual-Luciferase^®^ Reporter Assay System (Promega Corporation, Madison, WI, USA). The transcriptional activity was evaluated by the LUC/REN ratio. Each experiment included three biological replicates, and the data were statistically analyzed to assess the regulatory effect of MdC3H49 on the *MdP5CS* promoter.

### 2.9. Transient Transformation Assay in Apple Calli

Uniformly growing apple calli were divided into a control group and a treatment group. The pCAMBIA-1302-GFP-MdC3H49 overexpression vector [33] was transiently transformed into the calli via *Agrobacterium*-mediated transformation [34]. After 2–3 days of dark culture, the calli were subjected to stress treatment. The treatment group was cultured in MS liquid medium supplemented with 4% PEG6000, while the control group was cultured in an equal volume of MS liquid medium without PEG6000. Both groups were incubated under identical conditions for 9 days. Morphological changes were observed and recorded to verify the transformation efficiency. Physiological indices related to drought response were measured to analyze the effect of MdC3H49 overexpression on drought tolerance in apple calli. Each experiment included three biological replicates, and the data were subjected to statistical analysis.

### 2.10. Gel Mobility Shift Assay (EMSA)

The EMSA technique was used to verify the binding ability of the MdC3H49 protein to the 5′-GGGAGA-3′ element in the MdP5CS promoter, with the specific steps as follows: Synthesize specific probes for the MdP5CS promoter containing the 5′-GGGAGA-3′ element (the forward sequence contains the 5′-GGGAGA-3′ core motif, and the reverse complementary sequence serves as a control), and biotin-label the forward probe. Perform prokaryotic expression and purification of the MdC3H49 recombinant protein, and verify the protein purity by SDS-PAGE electrophoresis. 3.Prepare the binding reaction system, including the purified MdC3H49 protein, biotin-labeled probe, and binding buffer, and set up a blank control (without protein), a competition group (adding 100-fold unlabeled probe), and a mutation group (probe containing the mutant 5′-GGGAGA-3′ element). After incubating the reaction system at 25 °C for 30 min, loading buffer was added and the binding products were separated by non-denaturing polyacrylamide gel electrophoresis. After electrophoresis, the gel was transferred to a nylon membrane, fixed by UV cross-linking, the probe signal was detected by chemiluminescence, exposed for imaging, and the binding bands were analyzed.

### 2.11. Transcriptome and Statistical Data Analysis

The sequencing data for transcriptome analysis were downloaded from the NCBI SRA Database (https://www.ncbi.nlm.nih.gov/ (accessed on 5 May 2025), SRA No. PRJNA1066126) [35], using the drought-tolerant genotype ZC-9 and drought-sensitive genotype Jizhen-2 as test materials. Raw sequencing data were filtered with the Trimmomatic (v0.39) to remove low-quality reads, adapter sequences and contaminated sequences, yielding clean reads. The clean reads were aligned to the reference genome using the Hisat2 software (v2.2.1) [36], and gene expression levels were quantified by the StringTie software (v2.2.1) [37] and represented as FPKM values. Differential expression analysis was performed with the DESeq2 software (v1.38.3) [38], and differentially expressed genes (DEGs) were identified with the screening criteria of |log2FC| ≥ 1 and padj < 0.05. All experimental data were statistically analyzed using SPSS 26.0 software. Quantitative data were expressed as “mean ± standard deviation”, and differences were tested by independent-samples *t*-test, with *p* < 0.05 indicating a significant difference and *p* < 0.01 indicating an extremely significant difference.

## 3. Results

### 3.1. Genome-Wide Identification and Chromosomal Location of the C3H Gene Family in Apple

This study used a combination of BLASTP and HMMER search methods to jointly identify the *MdC3Hs* in apples. After screening, a total of 85 candidate apple *MdC3H* genes were obtained. Subsequently, these genes were named *MdC3H1*~*MdC3H85* in sequence according to their positional order on the chromosomes. Statistical analysis of chromosomal locations (Figure S1) showed that among the 85 *MdC3H* genes, 15 were localized on chromosome 3, accounting for the largest number; followed by chromosomes 11 and 14, each with 8 localized genes; while chromosomes 8, 10, and 17 had the fewest localized genes, with only 3 each. Further analysis of the physicochemical properties of the proteins encoded by these genes (Table S1) revealed significant differences in protein length: the amino acid length ranged from 53aa (MdC3H21) to 2050aa (MdC3H79); the molecular weight ranged from 6.57 kDa to 225.14 kDa; the instability index distributed between 21.10 and 90.53; the isoelectric point (pI) ranged from 5.08 to 9.48; and the hydrophobicity index was all negative, indicating that these proteins are all hydrophobic. The above analysis of protein molecular characteristics indicated that there are certain differences among the MdC3H proteins in apples, which may lead to differences in their functions.

### 3.2. Evolutionary Divergence of the MdC3H Family Is Associated with CCCH-Type Zinc Finger Motif Features

**Figure 1:**
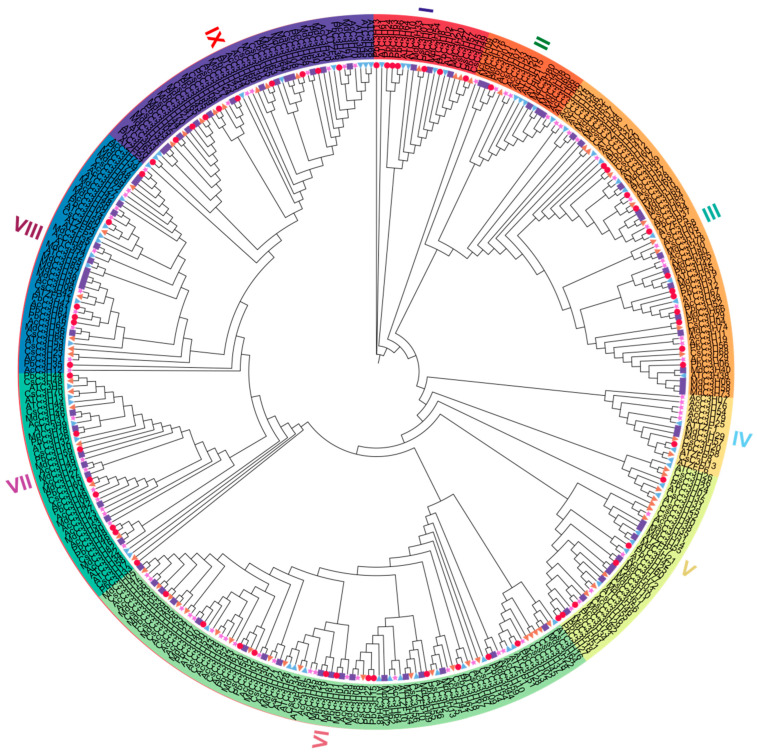
Phylogenetic analysis of the C3H protein family in apple. At. *Arabidopsis*; Cs. *Citrus sinensis*; Pb. *Pyrus bretschneideri*; Ac. *Actinidia chinensis*. The I–IX clades represent distinct subfamilies.

To further explore the phylogenetic relationship of apple C3H family proteins, this study used MEGA10 software to construct a phylogenetic tree of a total of 453 C3H proteins from apple (85), *Arabidopsis* (66), pear (66), citrus (70), and kiwifruit (81). According to the classification of the phylogenetic tree, the C3H proteins were divided into 9 subfamilies (I–IX) (Figure 1). Among them, subfamily VI contained the largest number of MdC3H proteins (17), while subfamily IV contained the fewest (only 2). Subsequently, statistical analysis of CCCH-type zinc finger motifs in the above five species (Table 1) revealed that apple C3H proteins covered all eight categories of CCCH-type zinc finger motifs. Moreover, apple possessed the largest total number of CCCH-type zinc finger motifs among the C3H family, reaching 199. Among them, the C-X_9_-C-X_5_-C-X_3_H type was the most common motif (102), followed by the C-X_12_-C-X_5_-C-X_3_H type (58). Comparison among the five species found that the C-X_4_-C-X_5_-C-X_3_H type was absent in apple and kiwifruit but present in the other three species; the C-X_7_-C-X_5_-C-X_3_H type was identified once each in apple, kiwifruit, and *Arabidopsis*, but absent in the other two species. In summary, these results reflect the differences in evolutionary characteristics of CCCH-H type genes.

### 3.3. Conservation and Gene Structure Divergence of the MdC3H Family Indicate Functional Diversification

**Figure 2:**
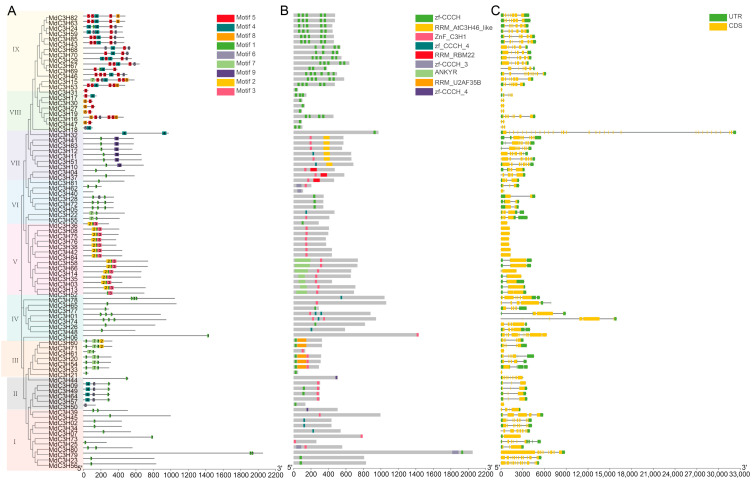
Analysis of conserved motifs (**A**), conserved domains (**B**), and gene structures (**C**) of the C3H protein family in apple.The I–IX clades represent distinct subfamilies.

To further explore the conservation of apple MdC3H family proteins, this study comprehensively analyzed the phylogeny, motifs, and domains of apple MdC3H family proteins (Figure 2). Nine conserved motifs of the apple MdC3H protein family were predicted, and it was found that the number of motifs of apple MdC3H family proteins varied greatly. According to the characteristics of conserved motifs of each protein, 85 proteins could be divided into 9 subgroups, and the motif composition and protein domains in each subgroup were very similar. Among them, the MdC3H19 protein had the most conserved motifs (7) (Figure 2A,B). In addition, except for MdC3H26, all the other 84 MdC3H proteins contained motif2. Further analysis of conserved domains found that all proteins in the apple MdC3H protein family contained CCCH domains, but the number of CCCH domains in each protein was different. Among them, 48.23% of the proteins contained the typical ZF-CCCH domain, and the remaining proteins contained atypical CCCH domains. Analysis of gene structure found that the *MdC3H32* gene had the longest sequence, containing only one CCCH domain at the C-terminus, and its gene structure was also very complex, with 32 exons, suggesting that its gene structure had mutated during evolution (Figure 2C). Overall, the CDS and UTR structural variations in apple *MdC3H* genes were consistent with their phylogenetic classification, indicating functional divergence among CCCH family members.

### 3.4. Collinearity Analysis Revealed That Apple Is Closely Related to Malus sieversii and Actinidia chinensis During Evolution

**Figure 3:**
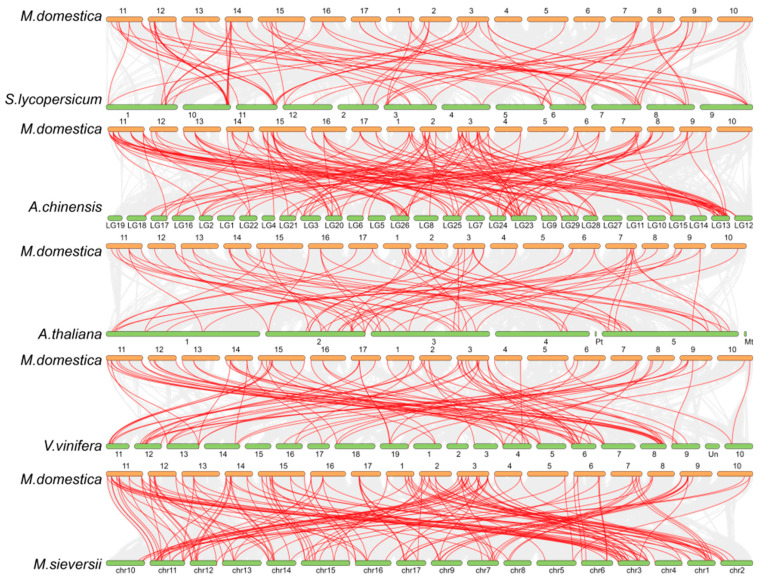
Collinearity analysis of MdC3Hs between *M. domestica*. and *Solanum lycopersicum*, *Actinidia chinensis*, *Arabidopsis*, *Vitis vinifera*, and *Malus sieversii*. Numbers in the figure represent chromosome IDs.

By analyzing the collinearity of the *C3H* gene family in apples, the possible evolutionary relationships and potential duplication events of *C3H* genes were explored. Blastp software(v.2.16.0) was used for sequence alignment, and MCScanX software(v.1.0) was used for multiple collinearity analysis to construct interspecific collinearity maps between apples and *Arabidopsis*, tomato, kiwifruit, grape, and *M. sieversii* (Figure 3). The results showed that *Malus sieversii* and apple had the largest number of collinear gene pairs (150), indicating the closest evolutionary relationship between them; followed by 141 collinear gene pairs between apple and kiwifruit In addition, in the collinearity map between apple and kiwifruit, chromosome 13 had the most collinear gene pairs (18); homologous genes of apple C3Hs were found in both kiwifruit and *M. sieversii*, indicating high homology between apple and kiwifruit, as well as between apple and *M. sieversii*. Apple had the fewest collinear gene pairs with *Arabidopsis* (only 72), second only to 77 pairs between apple and tomato. It can be seen that during the evolution of *C3Hs* from *Arabidopsis* to apple, the number of family genes showed a gradual expansion trend.

### 3.5. Analysis of Cis-Acting Elements in the Promoters of MdC3Hs

**Figure 4:**
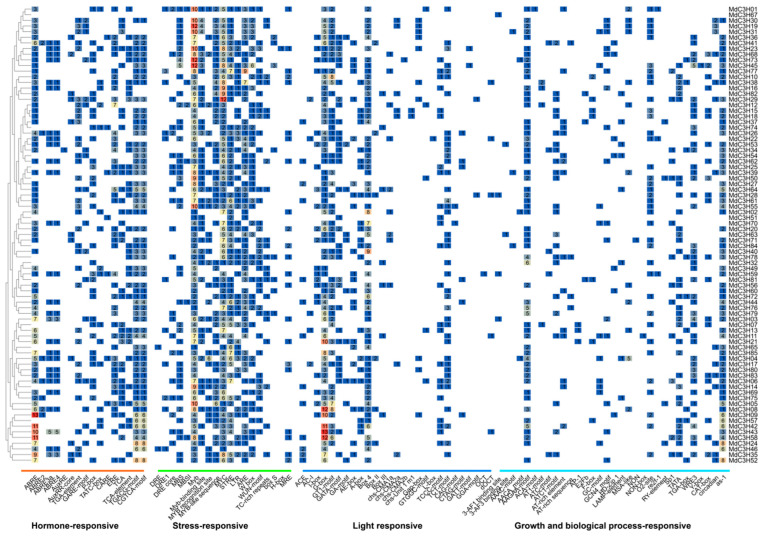
Distribution and abundance of cis-acting elements in the promoter regions of 85 *MdC3Hs* in apple. Values in the heatmap indicate the number of cis-acting elements.

The 2000 bp promoter sequences upstream of 85 *C3H* genes were extracted from the apple genome, and their cis-acting elements (CAEs) were predicted through the PlantCARE website. The results showed that a total of 1518 CAEs were predicted, which could be divided into 17 types and 4 functional modules: light response, stress response, hormone response, and plant growth and development response elements (Figure 4). Among them, light response elements included G-box, GT1-motif, Box4, ATC-motif, and ACE; stress response elements included low-temperature stress (LTR), anaerobic induction (ARE, GC-motif), drought stress (MBS), and wound-responsive element (WUN-motif); hormone response elements included auxin (TGA-element, AuxRR-core), gibberellin (TATC-box, P-box, GARE-motif), abscisic acid (ABRE), methyl jasmonate (TGACG-motif, CGTCA-motif), and salicylic acid response element (TCA-element); plant growth and development response elements included circadian rhythm control (circadian), flavonoid biosynthesis gene regulation (MBSI), and meristem-related (CAT-box) cis-acting elements. Statistical analysis of the prediction results found that among the 4 functional modules, compared with the light response and growth and development response modules, the stress response module and hormone response module had the largest number of CAEs although not the most types, indicating that the *C3Hs* in apples mainly play a role in stress response. In addition, in the hormone response module, all 85 *MdC3H* genes contained ABA response element ABRE or jasmonic acid-related element TGACG-motif, suggesting their important role in the stress resistance process. At the same time, the study found that the promoter regions of all 85 apple *C3H* genes contained light response elements, among which the I-Box element accounted for the highest proportion (59.5%); in the plant growth and development response module, the as-1 element accounted for the largest proportion (33.8%). The above results indicate that the apple *C3H* gene family may play an important role in responding to light signals, hormone signals, stress, and regulating apple growth and development.

### 3.6. GO Annotation of the MdC3H Family Genes

**Figure 5:**
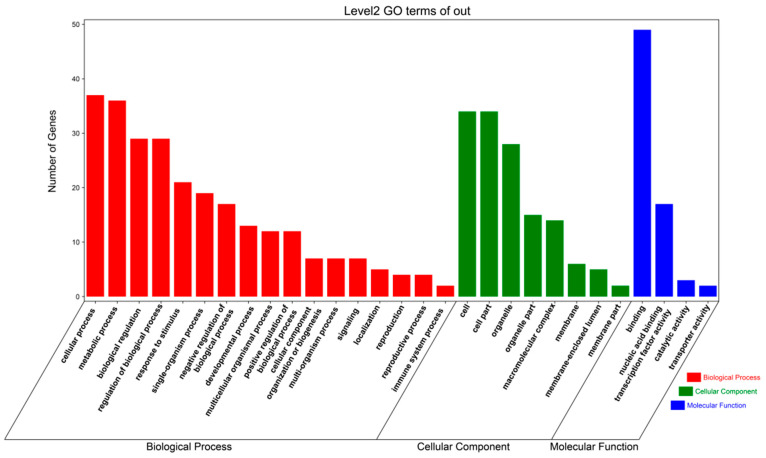
GO functional enrichment analysis of apple *MdC3H* genes. The figure shows the top 29 enriched GO terms in three categories (Biological Process, Cellular Component, and Molecular Function). The *x*-axis represents the number of genes, and the *y*-axis represents the name of GO terms.

To further conduct functional enrichment analysis of the 85 *MdC3Hs*, this study performed GO annotation on the family genes (Figure 5). The results showed that the enrichment results could be divided into three categories: Biological Process, Cellular Component, and Molecular Function. In the Biological Process category, “Cellular Process”, “Metabolic Process”, and “Biological Regulation” were significantly enriched, indicating that the *MdC3Hs* play an important role in cell metabolism regulation; in the Cellular Component category, the *MdC3Hs* mainly function in “Cell” and “Cell Part”; in the Molecular Function category, they are mainly involved in nuclear binding and transcriptional activation processes. In summary, the *MdC3Hs* play an important role in regulating cell functions.

### 3.7. MdC3H49 Is a Key Regulator Involved in Drought Stress Responses in Apple

**Figure 6:**
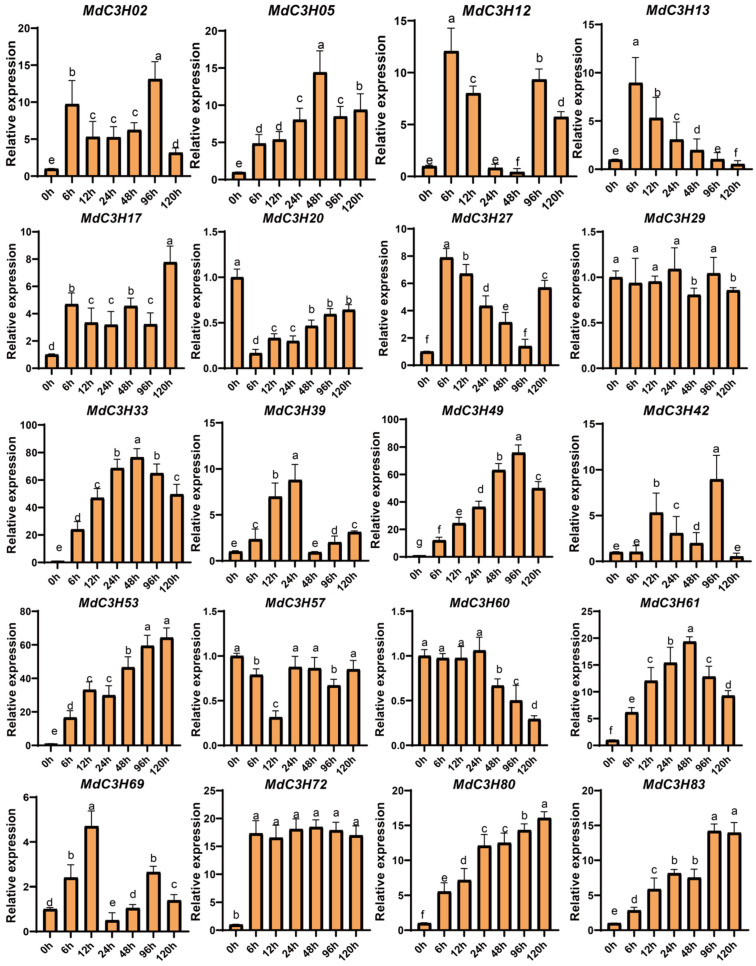
RT-qPCR analysis of the expression of 20 *MdC3Hs* under drought stress. The relative expression levels of target genes were detected by RT-qPCR, with *Actin* gene used as the internal reference to normalize the expression data. Different lowercase letters above the bars indicate significant differences among groups (*p* < 0.05) as determined by LSD method.

Under drought stress, the expression levels of different *MdC3H* genes in apple exhibited a significantly differential regulation pattern (Figure S2). In this study, at least one representative gene was selected from each subfamily. A total of 20 genes were used for quantitative expression analysis (Figure 6). The results showed that the expression of genes such as MdC3H02 and *MdC3H05* was increased under stress (*p* < 0.05), with an overall low-to-moderate upregulation level. *MdC3H12* and *MdC3H13* showed synchronous expression changes. They displayed a moderate upregulation trend and similar expression peak values. Genes including *MdC3H17* and *MdC3H20* exhibited weak expression activation with only slight fluctuations. The expression of *MdC3H27* and *MdC3H29* was significantly upregulated. These two genes were identified as highly responsive to drought stress. *MdC3H33* and *MdC3H39* showed moderate-to-high upregulation. *MdC3H49* displayed extremely strong expression induction. It was the most significantly drought-responsive member among all detected genes. *MdC3H42* also showed obvious upregulation. *MdC3H53* and *MdC3H57* exhibited similar upregulation trends. Both *MdC3H60* and *MdC3H61* were significantly drought-responsive genes with high expression increases. *MdC3H69*, *MdC3H72*, *MdC3H80*, and *MdC3H83* all showed varying degrees of upregulation. Among them, *MdC3H72* displayed relatively more prominent upregulation. Overall, most of the selected *MdC3H* genes were induced by drought stress. Only two genes, *MdC3H57* and *MdC3H29*, exhibited irregular expression changes in response to drought stress, suggesting that their induction by drought was minimal. The results indicated that five genes, including *MdC3H33*, *MdC3H49*, *MdC3H53*, *MdC3H61*, and *MdC3H83*, were most strongly induced by drought. To further identify the key drought-resistant gene among these five upregulated genes, we first performed cis-acting element analysis (Figure 4). We found that all five genes contained response elements for jasmonic acid (JA) and abscisic acid (ABA). Both JA and ABA are important regulatory hormones in drought stress responses [2,4]. Apple seedlings were then treated with JA and ABA. The expression levels of the five genes were subsequently measured. RT-qPCR analysis revealed that only *MdC3H49* was upregulated under both hormone treatments (Figure S3). We therefore speculate that *MdC3H49* functions as a core gene in the regulation of drought stress responses.

### 3.8. MdC3H49 Localizes to the Nucleus and Exhibits Transcriptional Activation Activity

**Figure 7:**
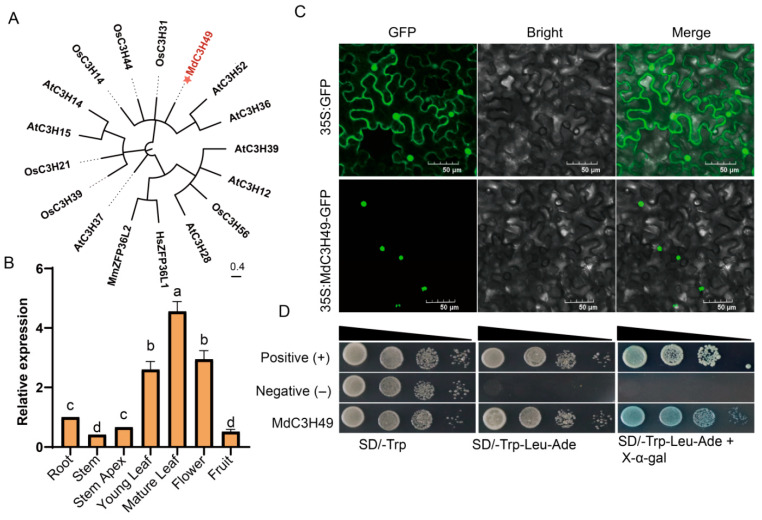
Tissue expression pattern and molecular characteristics of MdC3H49 protein. (**A**) Phylogenetic relationship between MdC3H49 and known homologous proteins; The orange text with star indicates the position of MdC3H49. (**B**) Expression characteristics of *MdC3H49* in different tissues; (**C**) Subcellular localization of MdC3H49; (**D**) Transcriptional activation activity analysis of MdC3H49. Statistical differences were analyzed using the LSD method. Different lowercase letters denote significant differences at *p* < 0.05; identical letters indicate no significant difference.

To further explore the molecular characteristics of the MdC3H49 protein, this study first performed a phylogenetic tree analysis of MdC3H49 together with known homologous proteins. The results showed that MdC3H49 is closely related to the AtC3H52 protein in *Arabidopsis thaliana*, suggesting that the two proteins may have similar functions. In addition, MdC3H49 was found to share a certain similarity with the mouse ZFP36L2 and human ZFP36L1 proteins, indicating that this protein is relatively conserved in the evolutionary process (Figure 7A). Further analysis of tissue-specific expression revealed that MdC3H49 had the highest expression level in mature leaves, while its expression levels in stems and fruits were the lowest (Figure 7B).To further analyze the subcellular localization characteristics of the MdC3H49 protein, this study used an *Agrobacterium*-mediated transient expression system in tobacco leaves for fluorescence microscopic observation. The experimental results showed that the control group (pCAMBIA1302-GFP empty vector) showed a typical whole-cell distribution pattern, with green fluorescent signals evenly distributed in the nucleus, cytoplasm, cell membrane and other regions; the experimental group (MdC3H49-GFP fusion protein) showed significant nuclear localization characteristics, with fluorescent signals specifically enriched in the nucleus (Figure 7C). Yeast transcriptional activation assay showed that yeast transformed with *MdC3H49* (coding sequence) could grow normally and turn blue on SD/-Trp-Leu-Ade medium containing X-α-gal, indicating that MdC3H49 has transcriptional activation function (Figure 7D). In summary, MdC3H49 is localized in the nucleus and has transcriptional activation activity.

### 3.9. Overexpression of MdC3H49 Significantly Enhances Drought Tolerance in Arabidopsis

**Figure 8:**
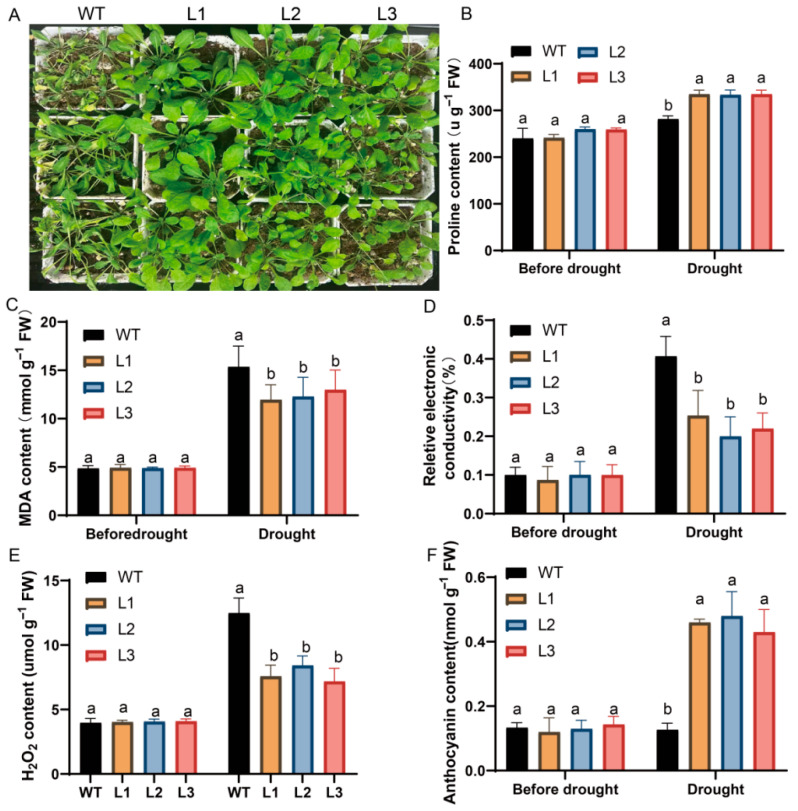
Phenotype and physiological indices of *MdC3H49*-overexpressing *Arabidopsis* under drought stress. (**A**) Phenotype of *MdC3H49*-overexpressing *A. thaliana* under drought stress; (**B**) Proline content; (**C**) MDA content; (**D**) Relative electrical conductivity; (**E**) Hydrogen peroxide content; (**F**) Anthocyanin content. LSD method was used to analyze differences between groups (*p* < 0.05), and different letters indicate significant differences between groups.

To further explore the function of the *MdC3H49* gene under drought stress, this study constructed *MdC3H49* ectopic overexpression *Arabidopsis* plants. Three-week-old *Arabidopsis* plants were selected for drought stress treatment, and after 11 days of continuous drought (Figure 8A), it was observed that the water loss and wilting degrees of the leaves of transgenic *Arabidopsis* were significantly lighter than those of the WT. Determination of proline content in *Arabidopsis* after drought stress found that the proline content of overexpression lines was significantly higher than that of the WT (Figure 8B). In addition, drought stress can lead to increased MDA and electrical conductivity in plants, which are two indicators that can directly reflect the degree of plant cell membrane damage; massive leakage of plant cell electrolytes is usually an important sign of damage to the cell membrane system. The determination results showed that compared with the WT, the relative electrical conductivity and MDA content of *MdC3H49* overexpression transgenic *Arabidopsis* were significantly reduced (Figure 8C,D), while the anthocyanin content was significantly increased, indicating that the degree of oxidative stress on *Arabidopsis* was significantly reduced (Figure 8E,F). Furthermore, the expression levels of six marker genes in *Arabidopsis* (*AtBAM4*, *AtP5CS*, *AtABI5*, *AtSOD*, *AtSAG21*, *AtNCED*) were detected. The results showed that, compared with the WT, the expression of all six genes was significantly up-regulated in the transgenic lines after drought treatment. In summary, this study confirmed that overexpression of the *MdC3H49* gene can significantly enhance the drought resistance of *Arabidopsis* by reducing cell damage caused by drought stress.

### 3.10. MdC3H49 Enhances Drought Resistance in Apple Calli by Regulating Physiological Indices and the Expression of Drought-Responsive Genes

**Figure 9:**
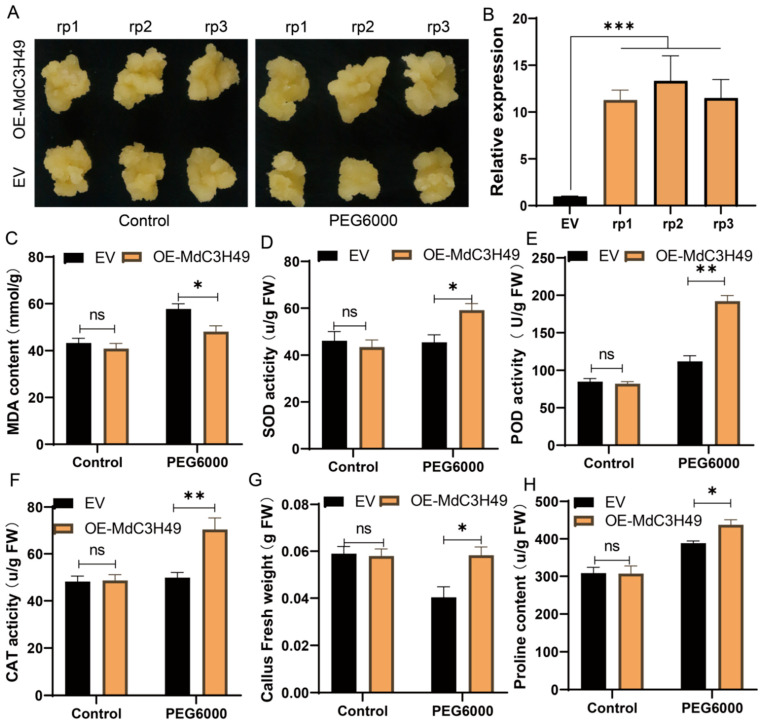
Transient transformation of *MdC3H49* in apple callus and determination of physiological indices under drought stress. (**A**) Apple callus treated with 15% PEG6000 to simulate drought stress; (**B**) Expression detection of *MdC3H49* in transiently transformed apple callus; (**C**) MDA content; (**D**) Superoxide dismutase (SOD) activity; (**E**) Peroxidase (POD) activity; (**F**) Catalase (CAT) activity; (**G**) Fresh weight of callus; (**H**) Proline content. Student’s *t*-test was used for comparative analysis. * *p* < 0.05; ** *p* < 0.01; *** *p* < 0.001. The rp1, rp2, and rp3 represent three independent biological replicates of the *MdC3H49*-OE lines (rp1: OE-rp1, rp2: OE-rp2, rp3: OE-rp3).

To further verify the regulatory effect of the *MdC3H49* gene on the drought resistance of apple calli, this study used ‘GL3’ apple calli as experimental materials to construct *MdC3H49* overexpression lines (*MdC3H49-OE*) and carried out drought stress-related experiments (Figure 9A). The results showed that under normal culture conditions, both the *MdC3H49-OE* transgenic lines and wild-type (WT) apple calli grew well, with no significant phenotypic differences; after drought stress treatment, the growth of both types of calli was inhibited to varying degrees, and the drought resistance of the *MdC3H49* overexpression lines was significantly stronger drought tolerance than that of the WT (Figure 9A,G). RT-qPCR detection results showed that *MdC3H49* was significantly overexpressed in the three overexpression callus lines (Figure 9B). To clarify the physiological mechanism by which *MdC3H49* regulates apple drought resistance, this study determined the relevant physiological indicators of apple calli under normal and drought stress conditions (Figure 9B). The results showed that under normal culture conditions, the damage degree of both *MdC3H49*-OE lines and WT was low. There were no significant differences in physiological indicators such as fresh weight between the two groups. Under drought stress, the MDA content of MdC3H49-OE lines was also significantly lower than that of the WT (Figure 9C). Detection of enzyme activities such as SOD, POD, and CAT found that these enzyme activity indicators were significantly higher in *MdC3H49-OE* lines than in WT (*p* < 0.05) (Figure 9D–F). In addition, detection of proline content found that the proline content of *MdC3H49-OE* lines was significantly higher than that of WT (*p* < 0.05) (Figure 9H). At the same time, this study also detected the expression levels of drought-related genes in apple calli under drought stress (Figure S5). The results showed that drought stress could significantly induce the expression of drought-related genes in apple calli, and under drought conditions, the expression levels of these genes in *MdC3H49-OE* lines were consistently higher than those in WT (Figure S5).

### 3.11. MdP5CS Is a Downstream Target Gene Regulated by MdC3H49

**Figure 10:**
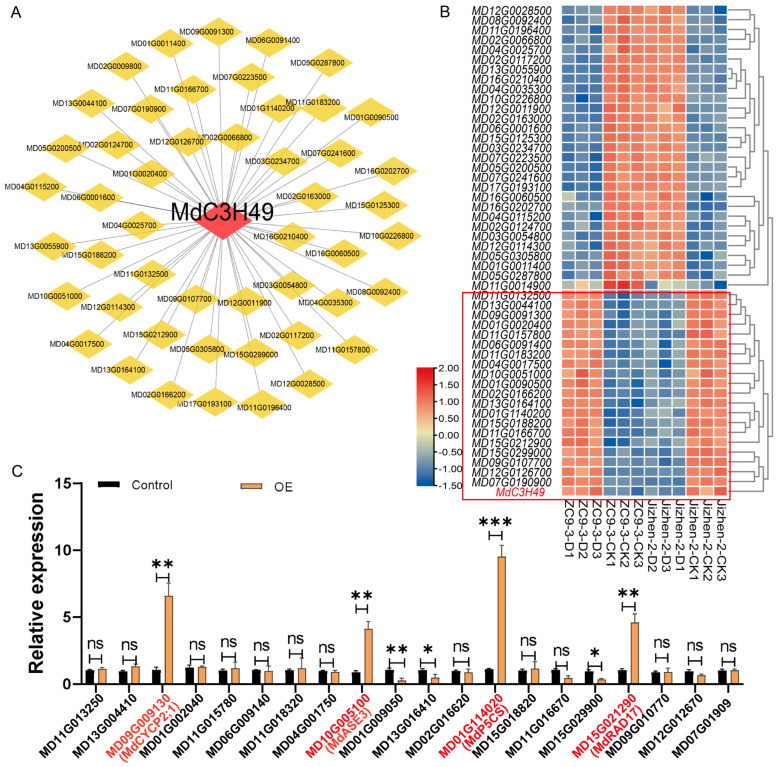
Screening of downstream regulatory genes of MdC3H49. (**A**) Prediction of potential regulatory genes of MdC3H49 based on WGCNA; (**B**) Expression pattern analysis of the top 50 candidate genes. The materials involved are the drought-tolerant genotype ZC-9-3 and the drought-sensitive genotype Jizhen-2. CK, control; D, drought treatment; (**C**) Expression detection of 20 candidate genes in *MdC3H49*-overexpressing apple callus. * *p* < 0.05; ** *p* < 0.01; *** *p* < 0.001. Red text denotes up-regulated genes; ns. No significant difference. The LSD method was used to analyze differences between groups (*p* < 0.05).

Weighted Gene Co-Expression Network Analysis (WGCNA) was employed in this study to predict candidate target genes associated with MdC3H49 to further explore the potential downstream regulatory target genes of the MdC3H49 protein. The CytoHubba plugin (a tool integrated with the network analysis software Cytoscape (v.3.10.0)) was used to identify key genes from the co-expression network generated by WGCNA. CytoHubba is specifically designed to identify and rank influential hub nodes within biological networks by leveraging various topological algorithms, which helps prioritize genes with critical regulatory roles. Following CytoHubba analysis, the top 50 genes with the highest correlation to *MdC3H49* were selected as initial candidate target genes (Figure 10A). Subsequent analysis of the expression trends of these 50 high-correlation genes revealed that 20 of them shared the same expression pattern as *MdC3H49* (Figure 10B).To validate these 20 candidate genes, RT-qPCR detection was performed in apple calli overexpressing *MdC3H49*. The results showed that the expression levels of 4 genes—*MdCYC2;1*, *MdASE3*, *MdP5CS*, and *MdRAD17*—were significantly higher than those in the control group (*p* < 0.05, Figure 10C). Further validation via GUS assay demonstrated that *MdC3H49* could only significantly activate the expression of *MdP5CS* (*p* < 0.05, Figure S6). Additionally, the expression trend of *MdP5CS* under drought stress was consistent with that of *MdC3H49* (Figure S7). Collectively, these results indicate that *MdP5CS* is the putative downstream regulatory target gene of *MdC3H49*.

### 3.12. MdC3H49 Interacts with the Promoter of MdP5CS and Activates Its Expression

**Figure 11:**
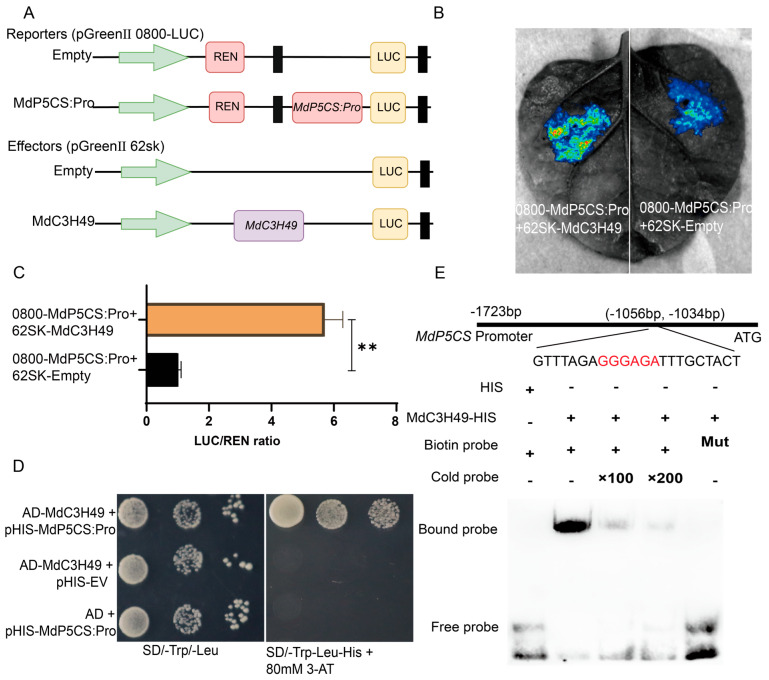
Verification that MdC3H49 binds to the *MdP5CS* promoter and activates its expression. (**A**) Schematic diagram of dual-luciferase reporter vector construction; (**B**) Dual-luciferase assay verifying the transcriptional activation of *MdP5CS* by MdC3H49; (**C**) Statistical analysis of LUC/REN ratios. Student’s *t*-test was used for comparative analysis. ** *p* < 0.01; (**D**) Y1H assay verifying the interaction between MdC3H49 and the *MdP5CS* promoter; (**E**) EMSA verifying the binding of *MdC3H49* to a specific cis-acting element in the *MdP5CS* promoter. Schematic diagram of the *MdP5CS* promoter region, with the core CCCH-binding motif (GGGAGAG, red) located at −1056 to −1034 bp upstream of the ATG start codon. The biotin-labeled wild-type (WT) probe and mutated (Mut) probe (with the core motif mutated) were used in the assay. Lane 1: Negative control (HIS tag only + biotin probe); Lane 2: MdC3H49-HIS fusion protein + biotin probe; Lanes 3 and 4: MdC3H49-HIS + biotin probe + 100× and 200× unlabeled cold competitive probe, respectively; Lane 5: MdC3H49-HIS + biotin-labeled Mut probe. The “Bound probe” indicates the protein-DNA complex, and “Free probe” indicates the unbound probe. The LSD method was used to analyze differences between groups (*p* < 0.05).

To further verify the interaction between MdC3H49 and *MdP5CS*, this study used a combination of multiple molecular biology experimental techniques for verification. First, the CDS of *MdC3H49* and promoter sequence of *MdP5CS* were constructed into pGreenII62sk and pGreenII0800-LUC, respectively, and then injected into tobacco. The results showed that MdC3H49 could significantly activate the expression of the *MdP5CS* promoter (Figure 11A,B). At the same time, this study used the pHis2 Y1H system to verify the interaction between MdC3H49 and the *MdP5CS* promoter (Figure 11C). Finally, EMSA assay showed that MdC3H49 could bind to a specific motif (5′-GGGAGA-3′) [39] on the *MdP5CS* promoter (3-AT, 80 mM) (Figure 11D). In summary, MdC3H49 can interact with the *MdP5CS* promoter and activate its expression.

## 4. Discussion

Apples originate from temperate regions, and their growth and development are vulnerable to drought stress. In arid and semi-arid producing areas such as northern China, frequent and long-lasting droughts severely affect fruit yield and quality [40,41]. Existing studies have revealed that the plant C3H gene family, as an important branch of the zinc finger protein family, participates in regulating various stress responses including cold tolerance and drought resistance, thereby alleviating damage to plants caused by low temperature and other adverse conditions [42,43]. In this study, a total of 85 MdC3H genes were identified in the apple genome, which is relatively high in number compared with those in plants such as *Solanum tuberosum* [44], *Glycine max* [12], and *Brassica rapa* [45]. It is speculated that this discrepancy may be jointly driven by apple-specific whole-genome duplication (WGD) events during evolution, as well as segmental duplication and tandem duplication events. This is consistent with the main causes underlying the expansion of most apple gene families (e.g., MADS-box and calcineurin B-like) [46,47]. According to the phylogenetic tree constructed based on *AtC3H* gene family members in *Arabidopsis thaliana* [7], the apple MdC3H gene family was classified into 9 subfamilies. Phylogenetic analysis between apple and Arabidopsis thaliana revealed that apple MdC3H members clustered into 5 subfamilies of the *AtC3H* gene family. MdC3H12, MdC3H28, MdC3H56, and MdC3H79 did not cluster with any *AtC3H* genes and formed an independent subfamily. In addition, homologs of AtC3H18 and AtC3H27 in *Arabidopsis thaliana* were not detected in apple.

Genome-wide identification showed that 85 *MdC3H* genes were obtained in apple, which were unevenly distributed on chromosomes, with the largest number on chromosome 3 and the smallest on chromosomes 8, 10 and 17. This uneven distribution pattern is consistent with the distribution characteristics of *C3H* gene families in other plants such as *Gossypium* [15] and *Oryza sativa* [8], indicating that the *MdC3H* gene family may have undergone uneven expansion during evolution, which may be related to chromosome duplication events. The physicochemical properties of MdC3H proteins varied significantly, with amino acid length ranging from 53aa to 2050aa and molecular weight varying greatly. And all proteins were hydrophobic. These differences suggest that MdC3H proteins may have different spatial structures and functional preferences, laying a foundation for their functional differentiation.

Phylogenetic analysis showed that 453 C3H proteins from 5 species were divided into 9 subfamilies, and MdC3H proteins were distributed in each subfamily, among which subfamily VI had the largest number of members and subfamily IV had the least. This classification is consistent with the evolutionary characteristics of the *C3H* gene family in other plants [12,13], indicating that the *C3H* gene family has been relatively conserved during long-term evolution, while there are also obvious species-specific differences. The statistical analysis of CCCH-type zinc finger motifs found that the C-X_9_-C-X_5_-C-X_3_H type was the most in apple, and there were differences in motif types among different species. For example, the C-X_4_-C-X_5_-C-X_3_H type was absent in apple and kiwifruit, which may be the key reason for the evolutionary divergence of the *C3H* gene family among species. In addition, Some family members contain additional conserved motifs, which is consistent with the structural characteristics of the C3H gene family in plants such as pear [48], rice [8], and wheat (*Triticum aestivum* L.) [49]. This indicates that C3H genes are highly conserved during plant evolution, with no obvious differentiation in their core functional domains. It is therefore speculated that their functions in regulating plant stress responses are conserved.

Collinearity analysis is an important means to explore the evolutionary relationship of gene families [50]. The results showed that apple had the most collinear gene pairs with *Malus sieversii*, followed by *A. chinensis*, indicating that apple had a close evolutionary relationship with these two species. *M. sieversii* is the ancestor of cultivated apple [51], and the high collinearity between the two species suggests that the *C3H* gene family has been relatively conserved during the domestication of apple, which may play an important role in maintaining the basic life activities of apple. The collinearity between apple and *Arabidopsis* was the weakest, and the number of *C3H* genes in apple was significantly more than that in *Arabidopsis*, indicating that the *C3H* gene family had an expansion trend during the evolution from dicotyledonous model plants to woody fruit trees, which may be related to the adaptation of apple to complex growth environments [52,53].

The analysis of cis-acting elements in promoters showed that the *MdC3H* gene family contained a variety of cis-acting elements related to light response, stress response, hormone response and growth and development, among which the number of stress response and hormone response elements was the largest, indicating that the *MdC3H* family mainly played a role in plant stress response. All *MdC3H* genes contained ABA- or jasmonic acid-responsive elements, suggesting a potential role for the *MdC3H* family in drought resistance via hormone signaling—a regulatory mechanism commonly observed in other plants, where homologous genes similarly respond to stresses and contribute to hormone-mediated adaptation [54]. GO annotation further confirmed that the *MdC3H* family was mainly involved in cell metabolism, nuclear binding and transcriptional activation, providing a direction for exploring the specific function of the *MdC3H* family.

Drought stress is one of the main abiotic stresses affecting apple growth and yield [28,55,56]. Screening key drought-responsive genes and clarifying their regulatory mechanisms are crucial for improving apple drought resistance. This study found that most *MdC3H* genes were induced by drought stress, among which *MdC3H49* had the most significant upregulation, especially in drought-tolerant apple varieties, indicating that *MdC3H49* is a key drought-responsive gene. Subcellular localization and transcriptional activation experiments showed that MdC3H49 is localized in the nucleus and has transcriptional activation activity, which is consistent with the characteristics of transcription factors [57], suggesting that MdC3H49 may regulate the expression of downstream target genes to participate in drought stress response.

Target gene prediction and verification showed that *MdP5CS* is a downstream regulatory target gene of MdC3H49. *MdP5CS* is a key gene in proline biosynthesis, and proline accumulation is an important way for plants to resist drought stress [58]. Y1H, Dual-Luc and EMSA experiments confirmed that MdC3H49 can directly bind to the specific motif (5′-GGGAGA-3′) on the *MdP5CS* promoter and activate its expression. This indicates that MdC3H49 may enhance plant drought resistance by promoting proline accumulation through regulating *MdP5CS* expression. Further functional verification in *Arabidopsis* and apple calli showed that overexpression of *MdC3H49* can significantly reduce MDA content and relative electrical conductivity, increase proline content and antioxidant enzyme activity, and upregulate the expression of drought-related genes, thereby enhancing drought resistance. This is consistent with the research results of *C3H* genes in other plants, such as *CpC3H3* in *Chimonanthus praecox* and *OsC3H10* in rice, which are also involved in the regulation of plant drought resistance [59], indicating that the regulatory role of *C3H* genes in drought stress is conservative among different species.

Although this study systematically explored the function and regulatory mechanism of the *MdC3H* family, there are still some limitations. First, the specific regulatory network of *MdC3H49* in apple drought stress response needs to be further explored, including other downstream target genes and interactive proteins. Second, the function of other *MdC3H* members in drought stress and other abiotic stresses (such as low temperature and salt stress) has not been verified, and the functional diversity of the *MdC3H* family needs to be further clarified. In addition, the role of *MdC3H49* in apple whole-plant drought resistance needs to be verified by stable transgenic apple plants.

## 5. Conclusions

In this study, 85 *MdC3Hs* were identified from apple via BLASTP and HMMER, and their chromosomal localization, physicochemical properties, phylogeny, conserved motifs, gene structure, collinearity, promoter cis-acting elements, and drought stress expression patterns were systematically analyzed. Phylogenetically, MdC3H proteins were divided into 9 subfamilies, with CCCH-type zinc finger motif differences causing evolutionary divergence between apple and other species. Conservation analysis showed high conservation of motif2 and widespread CCCH domains, with gene structural differences implying functional diversification. Collinearity analysis indicated apple’s closest evolutionary relationship with *M. sieversii*, followed by kiwifruit. Promoter cis-acting elements suggested *MdC3H* genes are involved in light, hormone, and stress responses. Notably, MdC3H49, a key drought-responsive factor localized in the nucleus with transcriptional activation activity, interacts with and activates its downstream target gene *MdP5CS*. Overexpression of *MdC3H49* significantly enhanced drought resistance in *Arabidopsis* and apple calli by regulating physiological indicators and drought-related gene expression. This study provides a theoretical basis for exploring *MdC3H*-mediated apple drought resistance and lays a foundation for drought-resistant molecular breeding.

## Figures and Tables

**Table 1:** Statistical analysis of conserved domain types in CCCH proteins.

Number	Type	Apple	Kiwifruit	Citrus	Pear	Arabidopsis
1	C-X_4_-C-X_5_-C-X_3_H	0	0	2	1	1
2	C-X_5_-C-X4-C-X_3_H	0	1	0	1	0
3	C-X_7_-C-X_6_-C-X_3_H	0	2	0	0	0
4	C-X_7_-C-X_5_-C-X_3_H	1	1	0	0	1
5	C-X_7_-C-X_4_-C-X_3_H	0	3	0	0	1
6	C-X_8_-C-X_6_-C-X_3_H	7	12	9	11	7
7	C-X_8_-C-X_5_-C-X_3_H	7	2	5	4	2
8	C-X_8_-C-X_4_-C-X_3_H	2	0	1	0	0
9	C-X_9_-C-X_5_-C-X_3_H	102	86	85	93	110
10	C-X_10_-C-X_5_-C-X_3_H	0	0	0	0	1
11	C-X_11_-C-X_5_-C-X_3_H	0	1	0	0	1
12	C-X_12_-C-X_5_-C-X_3_H	58	48	62	50	33
13	C-X_13_-C-X_5_-C-X_3_H	9	7	8	5	3
14	C-X_15_-C-X_5_-C-X_3_H	13	21	11	12	13
15	C-X_17_-C-X_6_-C-X_3_H	0	1	0	0	6
Total	C-X_4–17_-C-X_4–6_-C-X_3_H	199	185	183	177	179

## Data Availability

Data was used for the research described in the article.

## Supplementary Materials

The following supporting information can be downloaded at: https://www.mdpi.com/article/10.3390/plants15081270/s1, Supplementary Figure S1. Chromosomal localization analysis of MdC3H family genes in apple. Supplementary Figure S2. Expression pattern analysis of 85 MdC3H family genes in drought-tolerant (ZC-9) and drought-sensitive (Jizhen-2) apple cultivars. Supplementary Figure S3. Expression analysis of five genes under JA and ABA treatments. Supplementary Figure S4. Expression analysis of marker genes in Arabidopsis. Supplementary Figure S5. RT-qPCR validation of six marker genes from apple. Supplementary Figure S6. Verification of interactions between apple MdC3H49 and the promoters of MdCYC2;1, MdASE3, MdP5CS, and MdRAD17. Supplementary Figure S7. Expression characteristics of apple MdP5CS under drought stress. Supplementary Table S1. The information of apple C3H gene family genes. Supplementary Table S2. All the used Primers.
