# Supplementary material for: Genome-Wide Identification of the CCCH Gene Family and Functional Exploration of MdC3H49 Under Drought Stress Response in Apple (Malus domestica)
Source: Plants (Basel). 2026 Apr 21;15(8):1270. doi: 10.3390/plants15081270 (PMC13120152; doi:10.3390/plants15081270)
Supplement: Supplementary file 1 [file plants-15-01270-s001.zip › plants-4250967-supplementary/Figure Supplement.pdf]

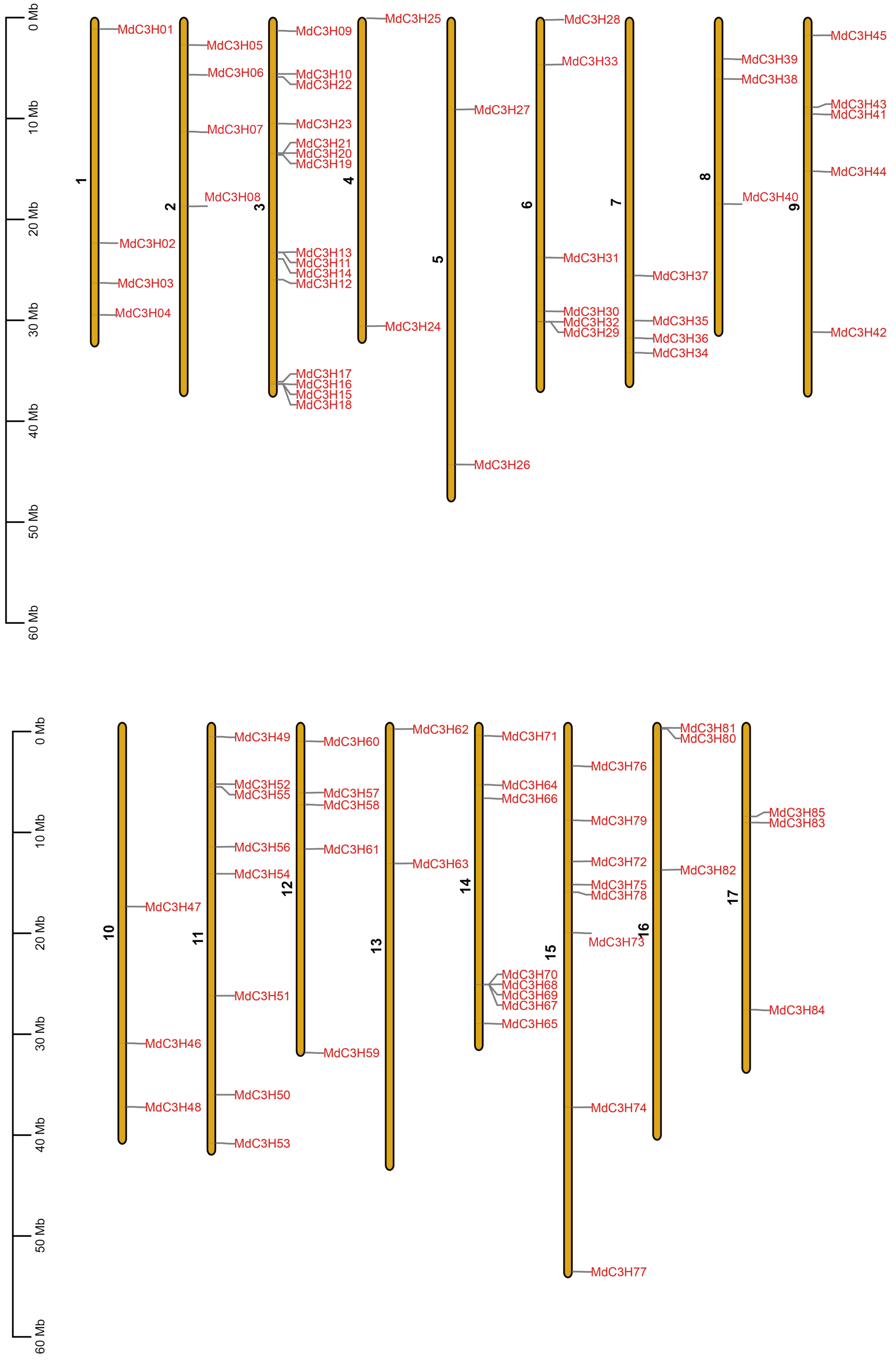

Fig.S1 Chromosomal localization analysis of MdC3H family genes in apple.

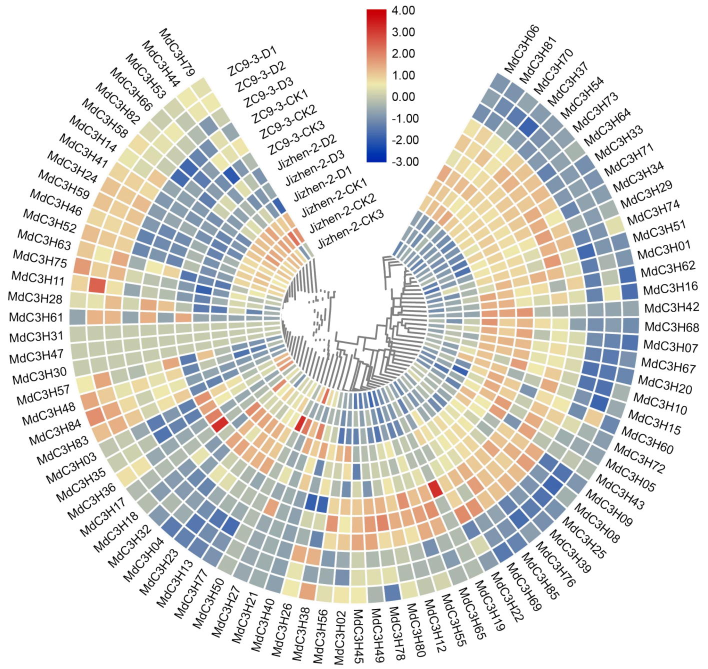

Fig.S2 Expression pattern analysis of 85 MdC3H family genes in drought-tolerant(ZC9-3) and drought-sensitive(Jizhen-2) apple cultivars.

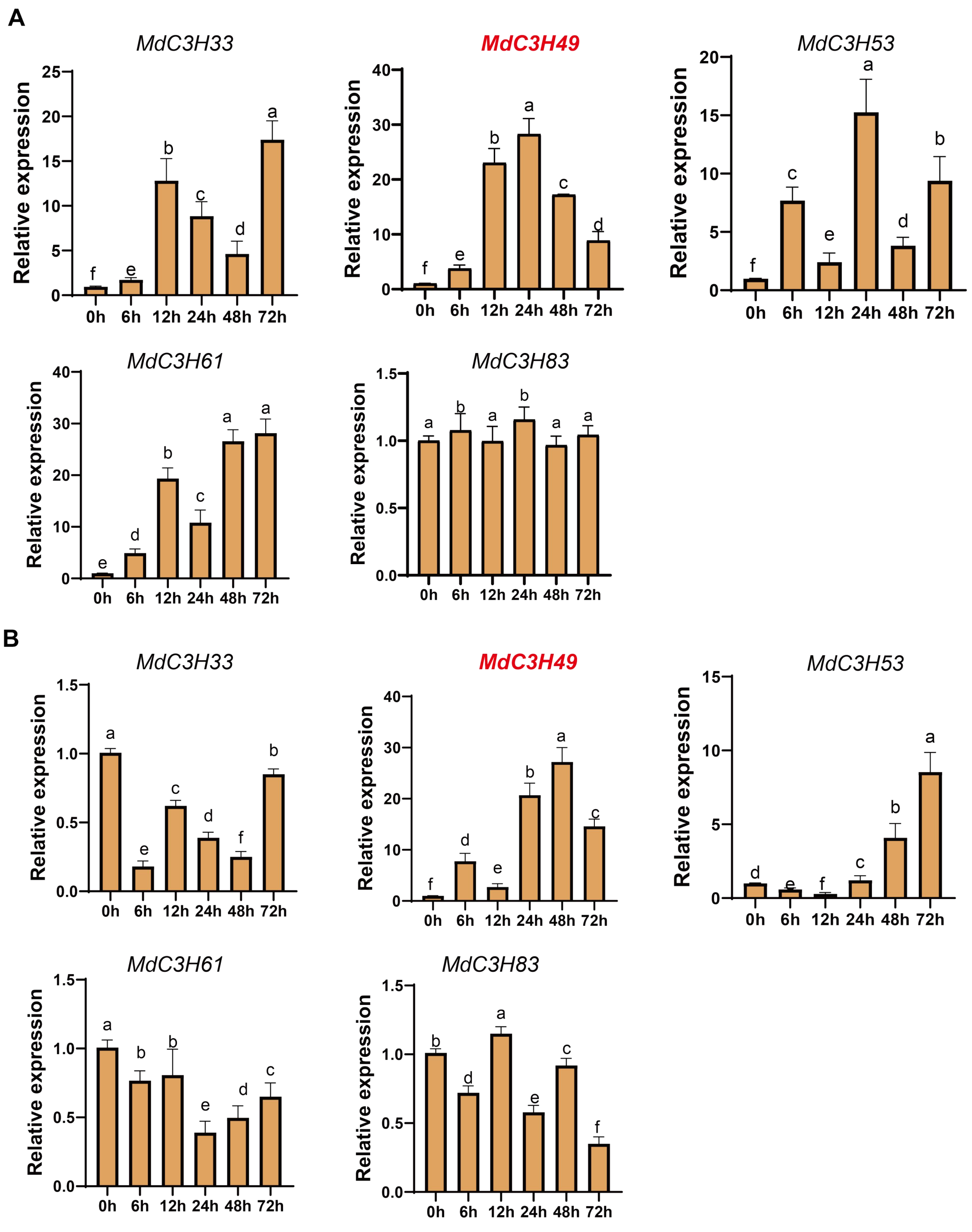

Fig.S3 Expression analysis of five genes under JA and ABA treatments. Significant differences were analyzed by LSD method (P < 0.05) (n=3). Different letters indicate significant differences.

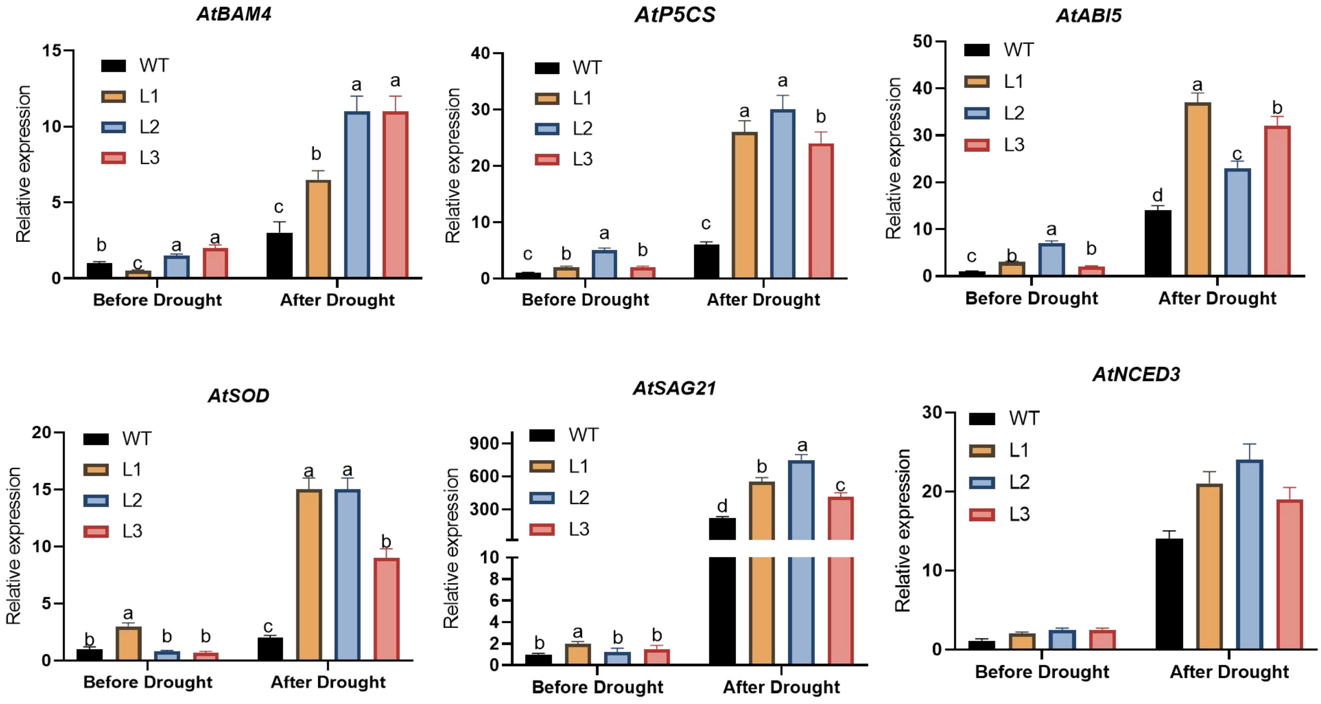

Fig.S4 Expression analysis of marker genes in *Arabidopsis*. The LSD method was used to analyze differences between groups ($P < 0.05$), and different letters indicate significant differences between groups.

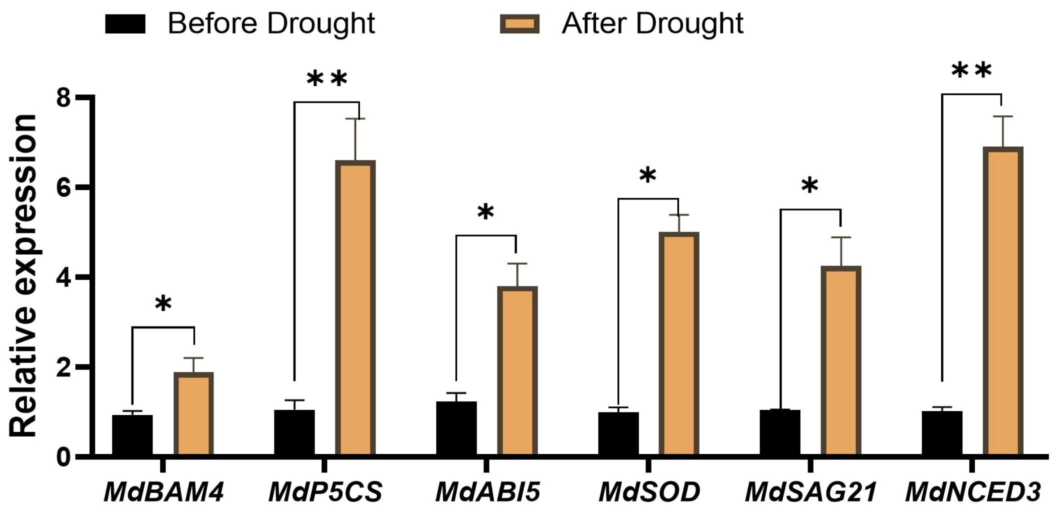

Fig.S5 qRT-PCR validation of six marker genes from apple.Statistical analysis was performed using Student’s *t*-test. *, $P < 0.05$; **, $P < 0.01$.

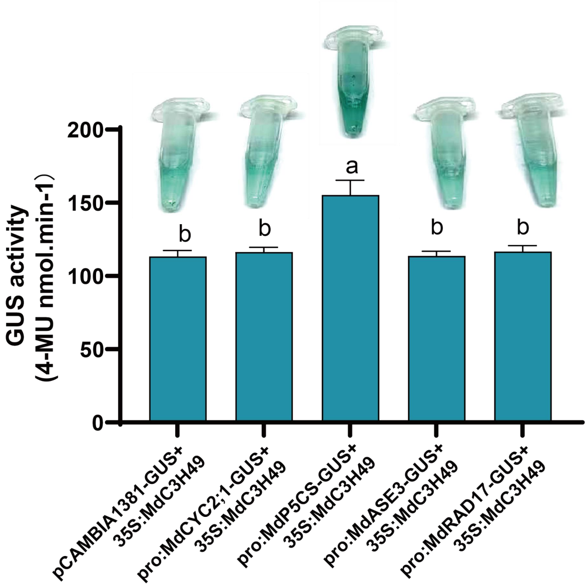

Fig.S6 Verification of interactions between apple MdC3H49 and the promoters of *MdCYC2;1*, *MdASE3*, *MdP5CS*, and *MdRAD17*.Differences among groups were analyzed by LSD test ($P < 0.05$) ($n = 3$). Different letters indicate significant differences.

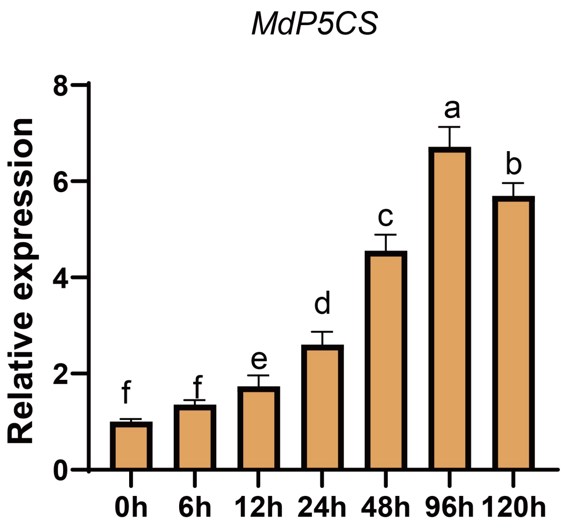

Fig.S7 Expression characteristics of apple MdP5CS under drought stress. Differences among groups were analyzed by LSD test (P < 0.05). Different letters indicate significant differences.
